# Special Issue “Molecular Mechanisms of Bioactive Nutrients Promoting Health Through Gut Microbiota 2.0”

**DOI:** 10.3390/ijms26031081

**Published:** 2025-01-26

**Authors:** Matteo Bordiga, Baojun Xu

**Affiliations:** 1 Department of Pharmaceutical Sciences, Università degli Studi del Piemonte Orientale “A. Avogadro”, Largo Donegani 2, 28100 Novara, Italy; matteo.bordiga@uniupo.it; 2 Food Science and Technology Program, Department of Life Sciences, BNU-HKBU United International College, Zhuhai 519087, China

This Special Issue of the *International Journal of Molecular Sciences* focuses on the highly relevant and rapidly developing topic of gut microbiota research. As the articles within this collection highlight, the human gut microbiota plays a critical role in human health. Dietary intake directly influences gut microbiota composition, and the resulting changes in gut metabolites can have profound effects on the host. This Special Issue brings together the latest findings on the intricate relationship between bioactive food components, gut microbiota, colon health, and chronic metabolic diseases.

The gut microbiota is a complex ecosystem composed of trillions of microorganisms, including bacteria, archaea, fungi, and viruses. These microbes play essential roles in digestion, nutrient absorption, immune function, and even mental health. Importantly, the composition of the gut microbiota is not static; it is constantly changing in response to diet, lifestyle factors, and medications.

A growing body of research suggests that bioactive components derived from dietary sources can be harnessed to modify gut microbiota composition and promote health. These bioactive components include phytochemicals (naturally occurring plant chemicals) and complex carbohydrates. However, the exact mechanisms by which these dietary components influence gut microbiota metabolism and how these changes affect human health remain unclear.

This Special Issue features a selection of articles that address these critical gaps in our knowledge. For instance, the article by Velderrain-Armenta et al. investigates the combined effects of *Bifidobacterium longum* and *Chlorella sorokiniana* on the antiviral cellular immune response [1]. Their findings suggest that this combination may be effective in boosting the immune system and protecting against rotavirus infection.

In another article by Mollace et al. explores the potential of bergamot polyphenolic extract combined with albedo and pulp fibers to counteract changes in gut microbiota associated with a high-fat diet [2]. Their study suggests that this combination may help to improve gut health and metabolic profiles in individuals with high-fat diet-induced dyslipidemia.

The article by Kato et al. takes a more holistic approach, examining the integrated multi-omics effects of fructo-oligosaccharide (FOS) supplementation on the human gut ecosystem [3]. Their findings highlight the significant inter-individual variability in response to FOS supplementation, suggesting the need for personalized approaches to prebiotic consumption.

Moving beyond the gut, Liu et al. explore the potential of egg-derived peptides to prevent obesity in a mouse model [4]. Their study suggests that these peptides may help to reduce lipid deposition and reprogram gut microbiota, ultimately leading to weight loss and improved metabolic health.

The complex interplay between diet, gut microbiota, and mental health is the focus of the article by Randeni and Xu [5], in which they review the latest evidence on how dietary components can influence gut microbiota composition and function, thereby impacting mood and reducing the risk of depression.

Randeni et al. also contribute another article to this Special Issue, this time exploring the triangular relationship between diet, gut microbiota, and inflammation [6]. Chronic inflammation is a key factor in many diseases, and this review highlights the potential of dietary interventions to modulate gut microbiota composition and reduce inflammation.

The article by Rodríguez-Daza and de Vos focuses on the role of dietary polyphenols in promoting the growth of *Akkermansia muciniphila*, a beneficial gut bacterium associated with improved gut health and metabolic function [7]. Their review explores the mechanisms by which polyphenols may exert these effects and the potential therapeutic implications.

Finally, Santhiravel et al. discuss the impact of plant phytochemicals on the gut microbiota [8], highlighting the potential of these bioactive compounds to modify gut microbiota composition and promote human health.

In conclusion, this Special Issue of the *International Journal of Molecular Sciences* provides a comprehensive overview of the latest research on the interplay between bioactive nutrients, gut microbiota, inflammation [9], and human health [10]. The research contributions to this Special Issue highlight the vast potential of dietary interventions to modulate gut microbiota composition and promote health. As our understanding of this complex ecosystem continues to grow, we can expect to see the development of novel therapeutic strategies for a wide range of chronic diseases. 
